# Effects of Component Prechilling and Ambient Temperature on the Handling and Thermal Characteristics of Two Polymethylmethacrylate (PMMA) Bone Cements

**DOI:** 10.7759/cureus.117784

**Published:** 2026-10-11

**Authors:** Szoradi Gergo, Andrei-Marian Feier, Octav M Russu, Sandor G Zuh, Tudor S Pop

**Affiliations:** 1 Department M4 Clinical Sciences, Orthopedics and Traumatology I, George Emil Palade University of Medicine, Pharmacy, Science, and Technology of Targu Mures, Mures, ROU; 2 Department M4 Clinical Sciences, Orthopedics and Traumatology I, George Emil Palade University of Medicine, Pharmacy, Science, and Technology of Targu Mures, Targu Mures, ROU

**Keywords:** arthroplasty, bone cement, polymerization temperature, polymethyl methacrylate, prechilling, setting time

## Abstract

Background and objectives: Polymethylmethacrylate (PMMA) bone cement remains a very important part of implant fixation in joint arthroplasty. Surgeons must find a way to balance the working time with the risk of excessive heat generated through polymerization that may contribute to thermal necrosis at the cement-bone interface. Handling and heat generation depend on the temperature of the cement components and the surrounding environment. This bench study examined component prechilling and ambient temperature in two arthroplasty cement formulations.

Methods: Sixteen batches of Simplex P (Stryker Corporation, Kalamazoo, MI, USA) and 16 batches of an antibiotic-loaded Simplex formulation were evaluated in a 2 × 2 temperature design: components conditioned at 21 °C or 4 °C and specimens cured at ambient temperatures of 21 °C or 16 °C. Cement was mixed manually for 30 s and formed into two 2-cm cylinders per batch. The recorded endpoints were an operational working-time measure, indentation-defined hardening time, and peak core temperature.

Results: With Simplex P, prechilling at an ambient temperature of 21 °C increased mean working time from 210.4 to 315.6 s and hardening time from 645.2 to 912.8 s, while peak temperature decreased from 78.4 to 62.1 °C. The combined 4 °C component and 16 °C ambient condition produced a working time of 398.1 s, a hardening time of 1054.3 s, and a peak temperature of 60.4 °C. Corresponding values for antibiotic-loaded Simplex were 228.1 s, 682.4 s, and 76.2 °C under baseline conditions and 418.5 s, 1098.6 s, and 59.1 °C under the combined-cold condition.

Conclusions: Lower component and ambient temperatures prolonged the handling and hardening intervals and reduced peak core temperatures in this bench model. Component temperature produced larger descriptive changes than the 5 °C difference in ambient temperature. The completed factorial ANOVA confirmed significant main effects for component temperature, ambient temperature, and formulation without significant interaction effects. Lower component and ambient temperatures prolonged handling and hardening intervals and reduced peak core temperatures in this exploratory bench model. However, given the small sample size, non-randomized testing sequence, subjective handling endpoint, and simplified cylinder geometry, these findings do not establish a clinical cooling protocol. Clinical recommendations require further confirmation using verified commercial products, clinically representative cement geometries, and direct interface-temperature measurements.

## Introduction

Polymethylmethacrylate (PMMA) bone cement remains a standard fixation material in cemented hip and knee arthroplasty and is also used for temporary spacers, osseous defect filling, and vertebral augmentation [1-4]. In arthroplasty, the cement fills irregularities between the prepared bone and implant and transfers load through mechanical interlock rather than chemical adhesion [2,3]. Its performance therefore depends on both the cured material and the handling window during which the cement can be introduced, pressurized, and protected from motion or fluid contamination.

Commercial PMMA cements are supplied as a polymer powder and a liquid monomer. Their principal constituents commonly include prepolymerized PMMA or copolymer beads, benzoyl peroxide initiator, a radiopacifier, methyl methacrylate monomer, an amine activator, and an inhibitor [1,2,5]. Mixing activates free-radical polymerization. The material passes through mixing, doughing, working, and hardening phases, but the terminology and endpoint definitions differ between laboratory standards, manufacturer instructions, and clinical practice [5-8]. International Organization for Standardization (ISO) 5833 defines setting time from the temperature-time curve, whereas surgeons often use tactile or indentation endpoints at the operative field [6]. The distinction matters when studies compare nominal setting times obtained by different methods.

Temperature is one of the strongest determinants of these transitions. Rheological experiments have shown faster viscosity development at higher temperatures, and kinetic models relate ambient temperature to the swelling, dissolution, initiation, and propagation processes that govern curing [7-9]. Initial component temperature also changes apparent viscosity and handling behavior [10]. Storage and equilibration experiments, clinical observations, and theater-temperature studies consistently show shorter setting intervals as temperature rises, although substantial variability remains at any single room temperature [11-13]. Vacuum mixing and chilling have also been investigated together, demonstrating that processing choices can affect handling and cured-cement properties [14].

Polymerization is exothermic. High temperatures measured within bulk cement do not directly equal the temperature at the bone-cement interface because heat transfer depends on cement volume and thickness, implant material, cancellous bone geometry, perfusion, and the initial temperature of each element. Experimental work established that thermal injury to bone depends on both temperature and exposure duration [15]. Human tissue analysis has shown reduced osteocyte viability adjacent to thicker cement mantles [16]. Bench and operative thermometry studies have reported pronounced differences between cement-core and interface temperatures, while computational models confirm steep spatial gradients during curing [17-19]. For this reason, a lower core peak is a material-level thermal outcome rather than direct evidence that osteonecrosis has been prevented.

Antibiotic loading can alter cement microstructure, porosity, mechanical behavior, drug release, and thermal characteristics. These effects depend on antibiotic identity, dose, particle properties, and whether the formulation is commercially premixed or hand blended [20-23]. Plain and antibiotic-loaded cements should therefore not be assumed to respond identically to temperature manipulation. Product identification is especially important because Simplex P (Stryker Corporation, Kalamazoo, MI, USA) without antibiotic content and antibiotic-loaded Simplex with tobramycin are distinct commercial formulations in relevant markets.

Direct comparisons of component prechilling and ambient cooling across plain and antibiotic-loaded Simplex formulations remain limited. Hypothermic manipulation has extended handling time in vertebroplasty models [24], but arthroplasty teams need formulation-specific information on the size of the delay and accompanying thermal change. The primary objective of this bench study was to compare working time, indentation-defined hardening time, and peak core temperature after conditioning cement components at 4 °C or 21 °C and curing the specimens at ambient temperatures of 16 °C or 21 °C. A secondary objective was to describe whether the two cement formulations showed different response patterns.

## Materials and methods

Study design

A controlled bench experiment used a 2 × 2 temperature design for each of two PMMA cement formulations. The component-conditioning factor had two levels (4 °C and 21 °C), and the ambient factor had two levels (16 °C and 21 °C). The four conditions were tested for Simplex and an antibiotic-loaded Simplex formulation. No patients, human tissue, animals, or animal tissue were involved. The experimental structure is summarized in Table 1 and Figure 1.

**Table 1:** Experimental temperature conditions Note: Group sizes sum to 16 independent batches within each cement formulation; paired cylinders are averaged within batch. Simplex is manufactured by Stryker Corporation (Kalamazoo, MI, USA).

Condition	Components	Ambient	Simplex P batches	Antibiotic-loaded Simplex batches
Baseline	21 °C	21 °C	4	4
Prechilled	4 °C	21 °C	4	4
Cool ambient	21 °C	16 °C	4	4
Combined cold	4 °C	16 °C	4	4

**Figure 1:**
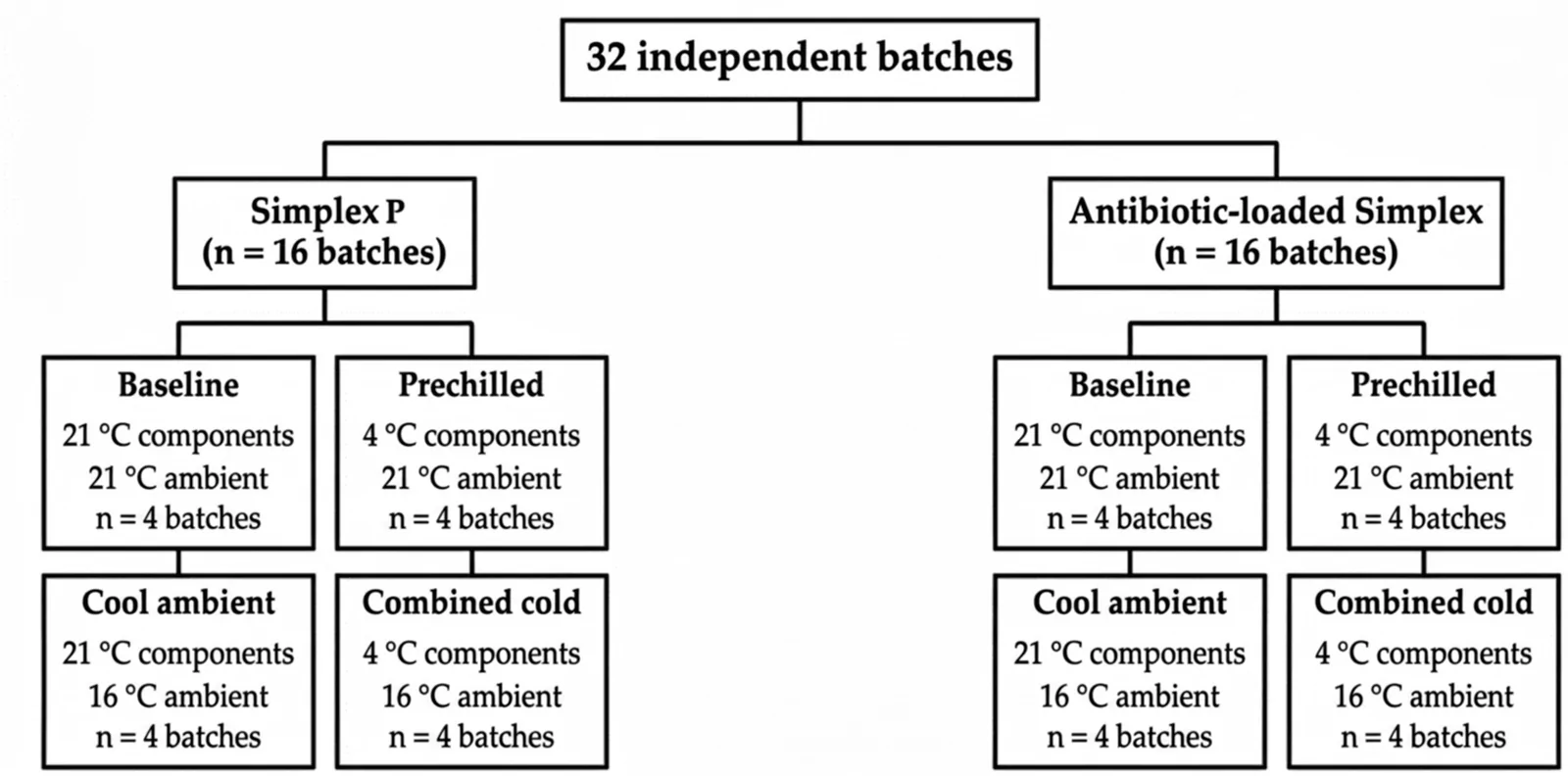
Factorial experimental design. Two measurements are averaged so that the batch remains the independent unit Simplex is manufactured by Stryker Corporation (Kalamazoo, MI, USA).

Testing took place in an environmentally controlled room. For workflow and temperature-equilibration reasons, the experiments were performed in a fixed, non-randomized sequence rather than in randomized order. Conditions were grouped into ambient-temperature blocks, with all 16 °C conditions tested before the 21 °C conditions, and within each block, components conditioned at 4 °C were tested before those conditioned at 21 °C. This sequence was selected to maintain stable environmental conditions and minimize repeated transitions between ambient-temperature settings during the experimental workflow. However, because experimental condition and testing sequence were therefore not independent, temporal effects, equipment drift, operator-related variation, or other unmeasured environmental changes occurring during the testing period could be partially confounded with the temperature conditions.

Cement formulations and experimental units

The experiment included 16 batches of Surgical Simplex P bone cement and 16 batches of Antibiotic Simplex with Tobramycin. Surgical Simplex P is supplied as a sterile two-component system containing 40.0 g of polymer powder and 20 ml of liquid methyl methacrylate monomer. Antibiotic Simplex cement was supplied as a commercially manufactured formulation containing 41 g of sterile white powder, containing 1 g of tobramycin and a 20 ml ampoule containing 19.5 ml of methyl-methacrylate, 0.5 ml N,N-dymethyl paratoluidine and 1.5 g of hydroquinolone. The manufacturer catalog number was 6191-0-001 for Surgical Simplex P and 6197-1-001 for Antibiotic Simplex with Tobramycin. The manufacturing lot numbers were CDH117 for Surgical Simplex P and 11LG092 for Antibiotic Simplex with Tobramycin. No additional antibiotic was manually incorporated by the investigators. The complete contents of each commercial cement unit were used for each experimental batch. No assumption of complete compositional equivalence between the two commercial formulations was made.

The full contents of each commercial unit were used for every experimental batch. For each cement formulation, independent batches were equally distributed across the four temperature conditions (n = 4 batches per condition). Two cylindrical specimens (2.0 cm diameter) were produced from each batch and measured independently. To avoid pseudoreplication, measurements obtained from the paired specimens were averaged before statistical analysis, with the individual cement batch serving as the independent experimental unit (N = 32 total batches).

Temperature conditioning

Unopened powder and monomer packages allocated to the prechilled conditions were stored for 24 h in a calibrated medical refrigerator at 4.0 ± 0.5 °C. Packages allocated to room-temperature conditions were stored for 24 h at 21.0 ± 0.5 °C. Mixing and curing were performed in a climate-controlled chamber at either 16.0 or 21.0 °C. Relative humidity was maintained at 45 ± 5%.

Mixing and specimen preparation

Timing began when the liquid and powder first contacted each other. A single operator manually mixed the full cement unit in a bowl with a spatula for 30 s. The dough was then divided and shaped into two cylinders with a nominal diameter of 2.0 cm. A K-type thermocouple was positioned at the geometric center of each cylinder and connected to a digital data logger with a stated accuracy of ±0.1 °C.

Mixing was performed manually by a single trained technician wearing non-powdered surgical gloves, using a stainless steel bowl (diameter, 12 cm; depth/capacity, 8 cm) and a flat stainless steel spatula for 30 s. Immediately following mixing, the dough was divided into two equal portions, manually shaped into cylindrical specimens (nominal diameter: 2.0 cm), and placed into silicone split molds within 45-60 s of mixing completion; excess cement was discarded. Central probe placement and outer specimen dimensions were verified using a custom mold alignment jig and confirmed post-curing via digital sliding calipers (accuracy: ±0.02 mm). A K-type thermocouple was positioned at the geometric center of each cylinder and connected to a digital data (accuracy: ±0.1 °C) recording at a sampling frequency of 1 Hz / 1 sample per second. Thermocouples were calibrated prior to testing using a two-point ice water (0 °C) and boiling water (100 °C) bath standard.

Outcome definitions

Operational working time was defined as the interval, measured in seconds from the initial contact between the liquid monomer and polymer powder, until the cement could no longer be manually shaped into the predefined cylindrical specimen without visible loss of structural integrity. The same trained operator performed the assessment using the same molding procedure for every batch. This study-specific endpoint was intended to quantify the practical handling interval under the experimental conditions and was not intended to represent the manufacturer-defined clinical working time or the ISO 5833 thermal setting time.

Hardening time was recorded from initial powder-liquid contact until a 2.0-mm indenter under a 49-N load no longer produced visible surface deformation. Indentation-defined hardening serves as a practical, physical endpoint measuring tactile surface resistance during cement application; while clinically relevant, it remains distinct from the ISO 5833 setting time. The ISO 5833 setting temperature was defined as the midpoint between the maximum temperature reached during polymerization and the recorded ambient temperature: Tmathrmset=(Tmax-Tmathrmambient)/2 where Tmathrmset is the setting temperature (°C), Tmax is the maximum temperature reached during polymerization (°C), and Tmathrmambient is the ambient temperature recorded at the beginning of the test (°C). The ISO 5833 setting time tmathrmset was defined as the elapsed time from the beginning of mixing until the temperature-time curve reached Tmathrmset. This thermal endpoint is distinct from the indentation-defined hardening time used as a separate operational outcome in the present study.

Peak core temperature was defined as the highest temperature recorded by the centrally positioned K-type thermocouple during polymerization and was expressed in degrees Celsius (°C).

Statistical analysis

Continuous outcomes are summarized as mean ± standard deviation. A three-way full factorial analysis of variance (ANOVA) was performed incorporating three fixed factors: component temperature (4 °C vs. 21 °C), ambient temperature (16 °C vs. 21 °C), and cement formulation (Simplex P vs. antibiotic-loaded Simplex), alongside their two-way and three-way interaction terms. All 32 independent batch observations were included in the finalized model. All statistical analyses were conducted in SPSS version 28.0 (IBM Corp., Armonk, NY, USA) with a two-sided significance threshold set at α= 0.05.

Continuous handling outcomes (working time, hardening time, and peak polymerization temperature) were recorded for each cement specimen. Before fitting the factorial ANOVA models, model assumptions were assessed using the model residuals rather than by relying solely on separate normality tests within each experimental group. Normality of the residuals was evaluated using the Shapiro-Wilk test and visual inspection of normal Q-Q plots. Homogeneity of variance was assessed by inspection of residual-versus-fitted-value plots and by comparison of residual dispersion across experimental conditions. Because each experimental condition contained only four independent batches, formal normality tests performed separately within individual groups were considered to have limited ability to detect departures from normality and were therefore not used as the sole basis for assessing model assumptions. Diagnostic plots were reviewed for evidence of substantial departures from normality, unequal variance, or influential observations.

Effect sizes for the ANOVA terms were expressed as partial eta-squared (η p2), which was interpreted as a partial effect-size measure quantifying the magnitude of each factor's effect relative to the variance remaining after accounting for the other terms included in the model. Partial eta-squared was not interpreted as the proportion of total outcome variance explained by the factor.

Data met assumptions of normality via Shapiro-Wilk testing (p > 0.05) and homogeneity of variances (Levene’s test, p > 0.05). A three-way full factorial ANOVA was performed incorporating three fixed factors: component temperature (4 °C vs. 21 °C), ambient temperature (16 °C vs. 21 °C), and cement formulation (Simplex P vs. antibiotic-loaded Simplex), alongside their two-way and three-way interaction terms. All analyses were conducted in SPSS version 28.0 with a two-sided significance threshold set at α = 0.05.

Because the experimental sequence was fixed and testing-order information was not prospectively recorded as an independent analytical variable, testing order could not be incorporated as a covariate or blocking factor in the factorial ANOVA. The available dataset therefore permits evaluation of the prespecified temperature and formulation effects but does not permit a statistically robust post hoc separation of treatment effects from potential temporal or batch-sequence effects. Accordingly, the inferential results should be interpreted as condition-associated differences within this controlled bench experiment rather than as estimates that are fully independent of testing sequence.

## Results

Under baseline conditions (21 °C component/21 °C ambient), plain Simplex P exhibited a mean working time of 210.4 ± 14.2 s, an indentation-defined hardening time of 645.2 ± 38.1 s and a peak core temperature of 78.4 ± 3.2 °C. Prechilling components to 4 °C at room temperature (21 °C) prolonged working time by 105.2 s and hardening time by 267.6 s, while reducing peak exotherm by 16.3°C.

Lowering ambient temperature alone to 16 °C extended working time by 74.8 s and hardening time by 167.3 s, while reducing peak temperature by 5.8 °C. The combined-cold condition (4 °C component/16 °C ambient) produced the longest working time (398.1 ± 22.4 s) and hardening time (1054.3 ± 52.1 s), and the lowest peak temperature (60.4 ± 2.1°C), representing an overall hardening delay of 409.1 s (6.82 min) and an 18.0 °C drop in peak temperature relative to baseline.

Antibiotic-loaded Simplex followed identical directional trends. Baseline values were 228.1 ± 12.8 s for working time, 682.4 ± 34.6 s for hardening time, and 76.2 ± 2.9 °C for peak core temperature. Under the combined-cold condition, working time reached 418.5 ± 21.0 s, hardening time reached 1098.6 ± 48.8 s, and peak temperature decreased to 59.1 ± 2.0 °C.

Across all four matched thermal conditions, antibiotic-loaded Simplex consistently exhibited slightly longer working times (+16.8 to +20.4 s) and hardening times (+35.3 to +44.3 s), as well as lower peak core temperatures (-1.3 to -2.2 °C) compared to Simplex P (Table 2).

**Table 2:** Handling characteristics and thermal properties across experimental conditions Comp: Component; Amb: Ambient. Simplex is manufactured by Stryker Corporation (Kalamazoo, MI, USA).

Cement Formulation	Outcome	21 °C Comp / 21 °C Amb	4 °C Comp / 21 °C Amb	21 °C Comp / 16 °C Amb	4 °C Comp / 16 °C Amb
Simplex P	Working time (s)	210.4 ± 14.2	315.6 ± 18.5	285.2 ± 16.1	398.1 ± 22.4
	Hardening time (s)	645.2 ± 38.1	912.8 ± 45.4	812.5 ± 41.2	1054.3 ± 52.1
	Peak temperature (°C)	78.4 ± 3.2	62.1 ± 2.8	72.6 ± 2.9	60.4 ± 2.1
Antibiotic Simplex	Working time (s)	228.1 ± 12.8	332.4 ± 17.2	302.8 ± 15.9	418.5 ± 21.0
	Hardening time (s)	682.4 ± 34.6	948.1 ± 42.3	848.2 ± 39.5	1098.6 ± 48.8
	Peak temperature (°C)	76.2 ± 2.9	60.8 ± 2.4	70.9 ± 2.7	59.1 ± 2.0

Three-way factorial ANOVA output

Component temperature exerted the primary effect across all three measured endpoints (p < 0.001), accounting for over 90% of the variance in working time (ηp2 = 0.933), hardening time (ηp2 = 0.926), and peak core temperature (ηp2 = 0.902). Ambient temperature also significantly affected working time (p < 0.001), hardening time (p < 0.001), and peak temperature (p = 0.002). These values indicate significant effects of component temperature within the fitted factorial models. They should be interpreted as partial effect sizes rather than as percentages of the total variance in each outcome explained by component temperature.

Cement formulation had a modest but statistically significant main effect on working time (p = 0.012) and hardening time (p = 0.029), but did not significantly alter peak core temperature (p = 0.130). No statistically significant two-way or three-way interaction terms were detected (p > 0.05 across all terms). For the Component x Ambient temperature interaction, the estimated interaction effect was 10.4 s (95% CI: -18.9 to 39.7 s, p = 0.476, ηp2= 0.026) for working time, 14.2 s (95% CI: -31.8 to 60.2 s, p = 0.534, ηp2= 0.020) for hardening time, and -2.1°C (95% CI: -4.4 to 0.2°C, p = 0.076, ηp2= 0.149) for peak core temperature. Because cell sample sizes were small (n = 4), these non-significant interaction terms indicate that any major non-additivity was not detected in this study, rather than establishing strict physical additivity (Table 3).

**Table 3:** Three-way analysis of variance (ANOVA) for handling outcomes Comp: Component; Amb: Ambient; Form: Formulation.

Outcome	Factor/ Interaction	F	df_1_	df_2_	p-value	Partial η^2^
Working time	Component temperature	277.88	1	24	< 0.001	0.921
	Ambient temperature	146.50	1	24	< 0.001	0.859
	Cement formulation	7.61	1	24	0.012	0.241
	Comp x Amb	0.53	1	24	0.476	0.022
	Comp x Form	0.01	1	24	0.943	0.000
	Amb x Form	0.02	1	24	0.895	0.001
	Comb x Amb x Form	0.02	1	24	0.889	0.001
Hardening time	Component temperature	248.98	1	24	< 0.001	0.912
	Ambient temperature	105.23	1	24	< 0.001	0.814
	Cement formulation	5.51	1	24	0.029	0.187
	Comp x Amb	0.40	1	24	0.534	0.016
	Comp x Form	0.01	1	24	0.919	0.000
	Amb x Form	0.01	1	24	0.909	0.000
	Comb x Amb x Form	0.03	1	24	0.873	0.001
Peak core temperature	Component temperature	183.44	1	24	< 0.001	0.884
	Ambient temperature	12.43	1	24	0.002	0.341
	Cement formulation	2.50	1	24	0.130	0.094
	Comp x Amb	3.51	1	24	0.076	0.128
	Comp x Form	0.10	1	24	0.755	0.004
	Amb x Form	0.01	1	24	0.904	0.000
	Comb x Amb x Form	0.01	1	24	0.904	0.000

## Discussion

Component prechilling and a lower ambient temperature prolonged both recorded time endpoints and reduced peak core temperature in each cement formulation. The largest descriptive change occurred when prechilled components were mixed and cured in the 16 °C environment. Relative to baseline, that condition added 6.82 min to hardening for Simplex P and 6.94 min for antibiotic-loaded Simplex, while reducing peak core temperature by 18.0 and 17.1 °C, respectively. Component prechilling produced a larger change than the 5 °C reduction in ambient temperature. Antibiotic-loaded Simplex consistently cured more slowly and at a slightly lower peak temperature than Simplex P.

The direction of these findings agrees with established PMMA rheology and polymerization kinetics. Farrar and Rose measured a strong temperature dependence of viscosity development between 19 and 25 °C [7]. Milner likewise showed that ambient temperature affects both polymer-particle swelling and the overall setting process [9]. Sullivan and Topoleski found that initial component temperature changes apparent viscosity and clinically relevant handling behavior [10]. The present results extend that pattern to a combined component-and-ambient design, and the completed factorial ANOVA quantifies the magnitude of each temperature effect alongside their interaction terms. No statistically significant interactions were detected between component chilling and ambient cooling, indicating that temperature reduction across both factors acted in a mutually supportive, non-interfering manner across both formulations.

Published clinical and laboratory data also show that room temperature alone cannot predict the exact curing interval. Langdown et al. found an inverse association between theater temperature and clinically assessed setting time for Simplex formulations but considerable variation at a given temperature [12]. Elliott et al. reported the same limitation in total knee arthroplasty [13]. Koh et al. showed that storage temperature and the time allowed for components to equilibrate affect doughing, working, and setting times [11]. Accordingly, a reproducible cement-temperature protocol requires control of component storage, transfer time, mixing equipment, ambient conditions, and the endpoint used to determine when loading is safe.

The magnitude of the handling extension is plausible in relation to prior hypothermic studies. Lidgren et al. combined chilling and vacuum mixing to alter cement handling and porosity [14], and Lai et al. prolonged the injectable period during vertebroplasty by cooling the cement and its container [24]. The current combined-cold means approached 6.6-7.0 min for the recorded working-time endpoint. The working-time values should be interpreted specifically as an operational handling endpoint generated by the present laboratory protocol. The endpoint was based on the ability to manually form the cement into the predefined specimen and therefore differs from both manufacturer-specific clinical working-time definitions and the ISO 5833 thermal definition of setting time. In particular, tactile or molding-based assessments can be influenced by operator judgment and may correspond more closely to a practical handling transition than to a standardized physicochemical setting endpoint. Consequently, the absolute working-time values should not be directly compared with manufacturer timing charts or studies using different endpoint definitions. The principal value of this measurement in the present study is the within-protocol comparison of the different temperature conditions. Loss of tack to a glove ordinarily marks doughing, whereas the end of working time occurs later when cement can no longer be applied or interdigitated according to the relevant product instructions [5,6,25]. Resolving this definition is necessary before comparing the values with manufacturer timing charts or other studies.

The thermal results require similar precision. Bulk PMMA can reach temperatures substantially above those measured at the bone-cement interface [17,18]. Harving et al. recorded a mean Simplex P core peak near 95 °C but a mean interface peak near 60 °C in an isolated-bone model [17]. In vivo hip measurements using modern cementing techniques reported lower average interface peaks [18]. Computational work identifies cement thickness, interface geometry, initial temperatures, and heat dissipation as major determinants of local exposure [19]. The 2-cm cylinders in this experiment intentionally concentrate material around the probe and do not reproduce a thin arthroplasty mantle. Their 59.1-78.4 °C core peaks therefore compare formulations and conditions within the model; they do not establish tissue temperature or thermal safety.

Experimental thresholds reinforce this distinction. Eriksson and Albrektsson demonstrated time-dependent bone injury after controlled heating in rabbits [15], and Whitehouse et al. found reduced human osteocyte viability adjacent to thicker PMMA mantles [16]. A lower core peak is directionally favorable, but the manuscript cannot claim that prechilling prevented osteonecrosis because no bone, viable cells, interface thermocouples, or time-above-threshold metric was studied. A clinically informative follow-up experiment should use a standardized cement mantle or cancellous-bone model, record the complete temperature-time curve at both core and interface locations, and calculate exposure duration above prespecified thresholds.

The modest formulation differences are compatible with evidence that antibiotic addition can change PMMA structure and curing behavior. Lewis et al. showed that the antibiotic blending method influences physical, mechanical, and thermal properties [20], and Dunne et al. documented simultaneous effects on strength, antibiotic release, and biofilm-related outcomes [21]. Reviews of antibiotic-loaded cement emphasize that the antibiotic, dose, commercial premixing process, and porosity all affect performance [22,23]. The present comparison, however, was between two distinct commercial cement formulations rather than between otherwise identical cements differing only by antibiotic addition. Therefore, the observed differences in hardening time and peak temperature cannot be attributed exclusively to tobramycin, because other formulation-specific compositional or manufacturing characteristics may also contribute. The results should consequently be interpreted as a comparison between the tested commercial products rather than as an isolated estimate of the effect of antibiotic incorporation.

These findings demonstrate the sensitivity of PMMA handling and polymerization dynamics to thermal conditioning within an exploratory laboratory setting. However, because this study evaluated 2-cm solid cylinders without surrounding tissue, fluid perfusion, or realistic mantle thickness, core peak reductions cannot be extrapolated to infer interface temperatures or thermal safety in total joint arthroplasty [25]. Consequently, these bench results do not establish or justify a specific clinical cooling protocol. Any clinical translation would require formal verification using standardized commercial products, anatomically representative cement mantle geometries, and precise thermometry at the cement-bone interface.

A recent experiment used warmed saline and an indentation endpoint to accelerate hardening of Simplex HV and PALACOS cement cylinders [26]. That work and the present cooling experiment address opposite methods of controlling the operative timeline, but neither indentation-based cylinder model is interchangeable with the ISO 5833 thermal definition. Together, they show that temperature manipulation can create several minutes of difference in an operational hardening endpoint. The practical choice should follow the product instructions and procedure requirements rather than a universal cold-or-warm protocol.

Limitations

Several methodological factors characterize this work as an exploratory bench study. The small number of independent batches (n = 4 per cell), non-randomized block testing sequence, subjective operational handling endpoint, and non-clinical cylindrical geometry prevent direct clinical extrapolation. The model lacked bone, implants, irrigation, physiologic heat dissipation, and interface thermometry. Therefore, these findings describe material-level behavior under controlled laboratory conditions rather than clinical outcomes or safety parameters. The indentation endpoint does not satisfy the ISO 5833 definition of setting time. The operational working-time endpoint was study-specific and depended on a manual handling assessment. Although the assessment procedure was standardized by using the same trained operator and the same molding procedure, the endpoint remains less objective than an instrument-based rheological or thermal measurement and may not correspond directly to the manufacturer-defined clinical working time. It should therefore be regarded as a comparative handling endpoint rather than a standardized measure of PMMA setting time.

Additionally, with four independent batches per condition, tests for interaction terms have limited statistical power to detect minor or moderate non-additive effects. The absence of statistically significant interaction terms should therefore not be interpreted as definitive proof that component prechilling and ambient cooling act strictly additively, but rather that no interaction reached the threshold of statistical significance within the power limits of this experiment.

Additionally, testing was performed using a fixed, non-randomized block sequence, with ambient-temperature conditions tested in separate blocks and component-temperature conditions tested in a fixed order within those blocks. This approach was selected to simplify environmental control and workflow but introduced potential confounding between experimental condition and chronological testing order. Consequently, temporal changes in equipment performance, operator performance, environmental stability, or other unmeasured batch-related factors could theoretically contribute to some of the observed between-condition differences. Because an independent testing-order or batch-sequence variable was not prospectively recorded, the available data do not permit a reliable post hoc adjustment for these effects. The magnitude and direction of any resulting bias therefore cannot be quantified from the present dataset. This limitation reduces the strength of causal inference regarding the temperature effects and should be considered when interpreting the factorial ANOVA results.

Manual mixing introduces potential operator-dependent variation, although a single trained technician performed all mixing procedures in this study to maintain consistency. The present experimental protocol did not record individual package lot numbers, operator blinding, or direct measurement of component temperatures at the exact instant of mixing. Additionally, thermal logging focused on peak core temperature; the time required to reach peak temperature and the cumulative duration above specific thermal thresholds were not recorded. Although the commercial products and antibiotic-containing formulation are identified, the study was not designed to isolate the independent effect of tobramycin from other formulation-specific differences between the two commercial cements. Accordingly, mechanistic conclusions regarding the contribution of the antibiotic itself should be avoided.

## Conclusions

In this bench model, prechilling PMMA cement components and reducing ambient temperature prolonged the recorded handling and indentation-defined hardening intervals and reduced peak core temperature in Simplex P and antibiotic-loaded Simplex. The combined-cold condition added approximately 6.8-6.9 min to hardening and reduced peak temperature by 17.1-18.0 °C relative to baseline. In this bench model, prechilling PMMA cement components and reducing ambient temperature prolonged the recorded handling and indentation-defined hardening intervals and reduced peak core temperature in Simplex P and antibiotic-loaded Simplex. The completed batch-level factorial ANOVA confirmed primary main effects for component and ambient conditioning across both formulations. In this exploratory bench model, prechilling PMMA cement components and reducing ambient temperature prolonged handling intervals and reduced peak core temperatures in Simplex P and antibiotic-loaded Simplex. While the completed factorial ANOVA confirmed significant main effects for component and ambient conditioning, the small batch sample size, non-randomized testing order, manual handling assessment, and non-anatomical specimen geometry mean these results cannot establish a clinical cooling protocol. Clinical recommendations remain premature and require further confirmation using verified commercial products, clinically representative geometries, and direct interface-temperature measurements.
